# Salidroside Regulates Inflammatory Response in Raw 264.7 Macrophages via TLR4/TAK1 and Ameliorates Inflammation in Alcohol Binge Drinking-Induced Liver Injury

**DOI:** 10.3390/molecules21111490

**Published:** 2016-11-09

**Authors:** Peng Sun, Shun-Zong Song, Shuang Jiang, Xia Li, You-Li Yao, Yan-Ling Wu, Li-Hua Lian, Ji-Xing Nan

**Affiliations:** 1 Key Laboratory for Natural Resource of ChangBai Mountain & Functional Molecules, Ministry of Education, College of Pharmacy, Yanbian University, Yanji 133002, China; sunpeng3001@aliyun.com (P.S.); szsong@ybu.edu.cn (S.-Z.S.); 2014001052@ybu.edu.cn (S.J.); lixiawo@163.com (X.L.); 2015001055@ybu.edu.cn (Y.-L.Y.); ylwu@ybu.edu.cn (Y.-L.W.); 2 Clinical Research Center, Yanbian University Hospital, Yanji 133002, China

**Keywords:** salidroside, inflammation, alcoholic liver injury, TLR4, TAK1

## Abstract

The current study was designed to investigate the anti-inflammatory effect of salidroside (SDS) and the underlying mechanism by using lipopolysaccharide (LPS)-stimulated RAW 264.7 macrophages in vitro and a mouse model of binge drinking-induced liver injury in vivo. SDS downregulated protein expression of toll-like receptor 4 (TLR4) and CD14. SDS inhibited LPS-triggered phosphorylation of LPS-activated kinase 1 (TAK1), p38, c-Jun terminal kinase (JNK), and extracellular signal-regulated kinase (ERK). Degradation of IκB-α and nuclear translocation of nuclear factor (NF)-κB were effectively blocked by SDS. SDS concentration-dependently suppressed LPS mediated inducible nitric oxide synthase (iNOS) and cyclooxygenase-2 (COX-2) protein levels, as well as their downstream products, NO. SDS significantly inhibited protein secretion and mRNA expression of of interleukin (IL)-1β and tumor necrosis factor (TNF)-α. Additionally C57BL/6 mice were orally administrated SDS for continuous 5 days, followed by three gavages of ethanol every 30 min. Alcohol binge drinking caused the increasing of hepatic lipid accumulation and serum transaminases levels. SDS pretreatment significantly alleviated liver inflammatory changes and serum transaminases levels. Further investigation indicated that SDS markedly decreased protein level of IL-1β in serum. Taken together, these data implied that SDS inhibits liver inflammation both in vitro and in vivo, and may be a promising candidate for the treatment of inflammatory liver injury.

## 1. Introduction

Inflammation is an early response to tissue injury and foreign pathogens, and then the normal tissue structure and function are recovered. A normal inflammatory response regulates expression of pro-inflammatory and anti-inflammatory proteins. During these inflammation responses macrophages are essential cells that bridged innate and adaptive immunity.

The transcription nuclear factor (NF)-κB is the key molecule in host defense and inflammatory responses against microbial and viral infections [1]. In an inactive status, NF-κB normally residues in the cytoplasm as a heterotrimer consisting of p50, p65 and IκB proteins. When NF-κB is activated by inflammatory stimuli, such as LPS, upstream IκB kinase (IKK) is phosphorylated and activated via upstream of TGF-β activated kinase 1 (TAK1) [2]. And then IκB is undergoing phosphorylation and degradation, while NF-κB is translocated from cytoplasm into the nucleus. Once there, NF-κB binds to DNA and induces further pro-inflammatory gene expression and inflammatory response [3,4]. Therefore, the inhibition of the NF-κB pathway may have a beneficial effect in anti-inflammation.

The mitogen-activated protein kinases (MAPKs) are a family of serine/threonine protein kinases, including c-Jun NH_2_-terminal kinase (JNK), extra-cellular signal-regulated protein kinase (ERK) and p38, playing an essential role in in the regulation of cellular responses to cytokines and stresses [5]. Activation of MAPKs signaling pathways leads to the synthesis and release of pro-inflammatory mediators by activated macrophages during the inflammatory response [6].

Using the in vitro model of RAW 264.7 macrophages, recent studies have suggested that LPS could stimulate inducible nitric oxide synthase (iNOS) and cyclooxygenase-2 (COX-2) through the ERK and JNK signaling pathway [7], and other reports proved the activation of MAPK and NF-κB by LPS [8,9,10]. The crosstalk between NF-κB and MAPK pathways in response to inflammatory signal regulates many genes in inflammatory response [11,12].

**Figure 1:**
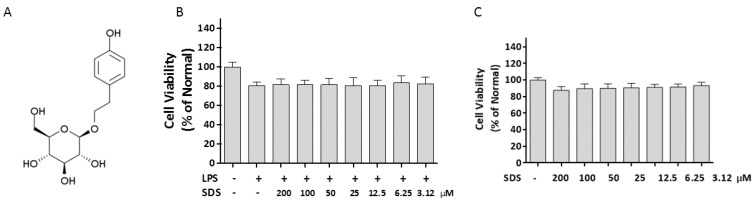
Effect of SDS on the cell viability. (**A**) Chemical structure of salidroside; (**B**) Cells were cultured with various concentrations of SDS for 24 h; (**C**) Cells were incubated with SDS in the presence of LPS (1 μg/mL) except normal group for 24 h. Cell viability was determined by MTT assay. Data are expressed as mean ± SD of three independent experiments.

Salidroside (SDS, *p*-hydroxyphenethyl-β-d-glucoside, structure shown in Figure 1A) is one of the active components of a highly valued medicinal herb called *Rhodiola sachalinensis,* which grows at high altitude and in cold regions, such as Changbai Mountain [13]. SDS displays a broad spectrum of pharmacological effects and has been used a long history of folk use [14]. It has been reported that SDS possesses various pharmacological properties including resisting anoxia, anti-aging, anti-cancer, anti-oxidative, hepatoprotective and cardioprotective effects [15,16]. In vivo, SDS could protect against acetaminophen-induced toxicity in C57BL/6 mice [17]. Recently Chang et al. reported SDS could alleviate ethanol-induced acute gastric ulcer and H_2_O_2_-induced gastric epithelial cell damage through the MAPK/NF-κB pathway [18]. Several references have reported that SDS regulated cytokine responses via NF-κB and ERK activation in LPS-stimulated RAW 264.7 macrophages in vitro and mice challenged with LPS in vivo [19,20]. However, there was no available research on the effect of SDS on inflammatory response on TLR4/TAK1 pathway in LPS-stimulated RAW 264.7 cells and binge drinking-induced liver inflammation. Thus the aim of the present study was to explore the anti-inflammatory mechanism of SDS in vitro and in vivo.

## 2. Results

### 2.1. Effect of SDS on Cell Viability

To examine the toxicity of SDS under experimental conditions, Raw 264.7 cells were treated with various concentrations of SDS. The cell viability was analyzed by MTT assay. LPS (1 μg/mL) alone only numerically but not statistically decrease the viability of RAW 264.7 cells (Figure 1B). SDS (200–3.12 μM) did not statistically affect RAW 264.7 cells viability in presence of LPS within 24 h (*p* > 0.5, Figure 1B). In addition, SDS alone displayed a slight concentration-dependent reduction of cell viability, especially at 200 μM, but there is no statistical difference compared to those of normal cells.

### 2.2. Effect of SDS on LPS-Induced iNOS and COX-2 Protein Expression

**Figure 2:**
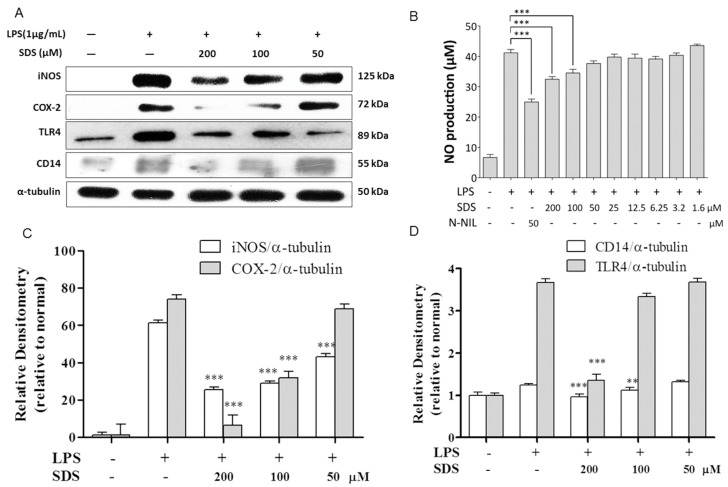
Effect of SDS on LPS-induced expression of iNOS, COX-2 protein and NO production. RAW 264.7 cells were pretreated with various concentrations of SDS for 1 h, followed by stimulating with LPS (1 μg/mL) for 24 h. (**A**) Representative pictures of Western blots and (**C**,**D**) quantitative analysis of blots. Each immunoreactive band was digitized and expressed as a ratio of α-tubulin levels; (**B**) NO level was measured by Griess reaction. L-NIL (N6-(1-iminoethyl)-lysine, hydrochloride, 50 μM) was used as positive control. Data are expressed as mean ± SD of three independent experiments. ** *p* < 0.01, *** *p* < 0.001, versus LPS-stimulated cells.

In the inflammation process, iNOS and COX-2 are pivotal enzymes in the inflammation process. We measured the protein levels of iNOS and COX-2 as well as production of NO in RAW 264.7 macrophages stimulated with LPS for 24 h. Protein expression of iNOS and COX-2 as well as NO release were minimal in normal cells, respectively (Figure 2A,B), but their levels were remarkably upregulated with LPS for 24 h. The induction of iNOS and COX-2 were dramatically suppressed by SDS pre-treatment in a concentration-dependent manner.

### 2.3. Effect of SDS on LPS-Induced TLR4 and CD14 Expression

The inhibition of iNOS and COX-2 expression by SDS in LPS-stimulated RAW 264.7 cells indicates that SDS influences the events in LPS signaling, such as the suppression of LPS binding to the cell surface receptors CD14/TLR4. Therefore, we tested whether SDS affects protein expression of TLR4 and CD14. As shown in Figure 2A, we found that protein expression of TLR4 and CD14 significantly increased in LPS-stimulated RAW 264.7 cells compared with normal cells. Pretreatment with 200 μM SDS significantly decreased the expressions of TLR4 and CD14, compared to those in LPS-stimulated cells.

### 2.4. Effect of SDS on LPS-Induced Phosphorylation of TAK1 and MAPK

**Figure 3:**
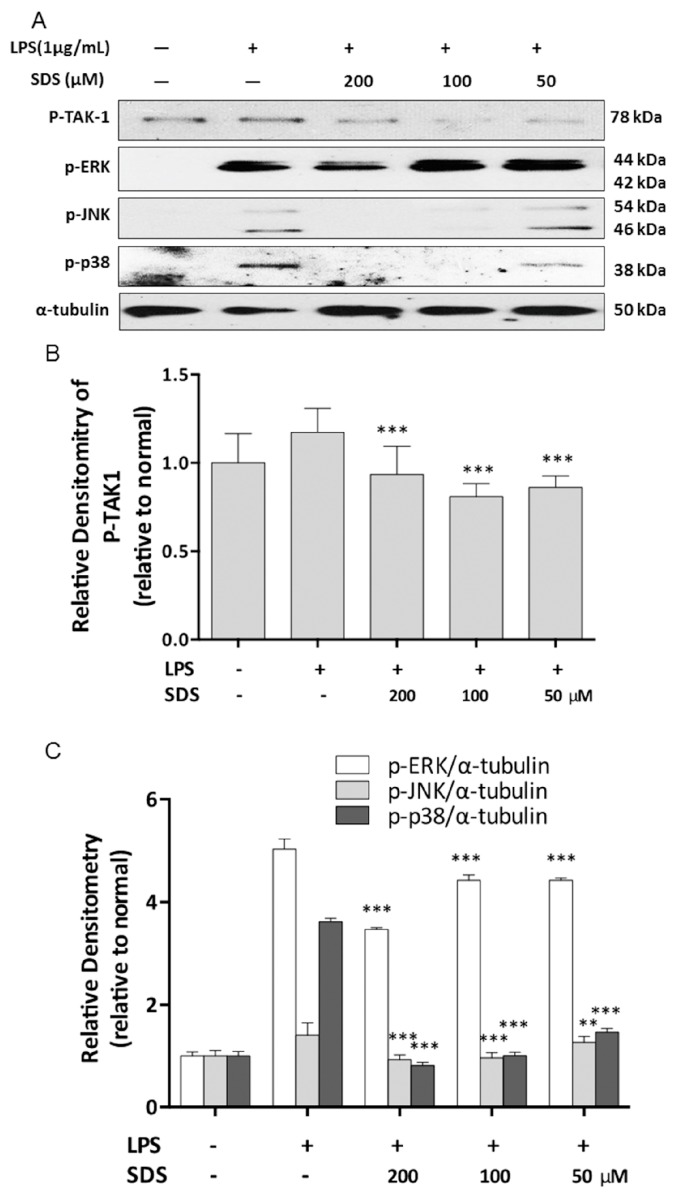
Effect of SDS on LPS-induced phosphorylation of TAK1 and MAPK. RAW 264.7 cells were pretreated with various concentrations of SDS for 1 h, followed by stimulating with LPS (1 μg/mL) for 30 min. (**A**) Representative pictures of western blots and (**B**,**C**) quantitative analysis of blots. Data are expressed as mean ± SD of three independent experiments. ** *p* < 0.001, *** *p* < 0.001, versus LPS alone.

TAK1 is the upstream signaling molecule of NF-κB, which regulates inflammatory genes such as COX-2, iNOS and proinflammatory cytokines. The effect of SDS on the LPS-induced phosphorylation of TAK1 was investigated using western blot. We found that an increase in p-TAK1 expression was detected with LPS for 30 min. However, pre-treatment with SDS inhibited this increase in TAK1 phosphorylation (Figure 3A).

MAPKs are involved in the regulation of pro-inflammatory mediator expression in LPS-treated RAW 264.7 cells [21]. We were analyzed the phosphorylation of three MAPK signaling molecules including ERK, JNK, and p38 MAPK. Our results confirmed that phosphorylation of ERK, JNK, and p38 MAPK were significantly increased by treatment with LPS. Pretreatment with SDS inhibited the Wephosphorylation of ERK only at a higher concentration of 200 μM, and markedly prevented the LPS-induced increasing of JNK and p38 phosphorylation in a concentration-dependent manner (Figure 3A).

### 2.5. Effect of SDS on LPS-Induced IκB Degradation and NF-κB Nuclear Translocation

**Figure 4:**
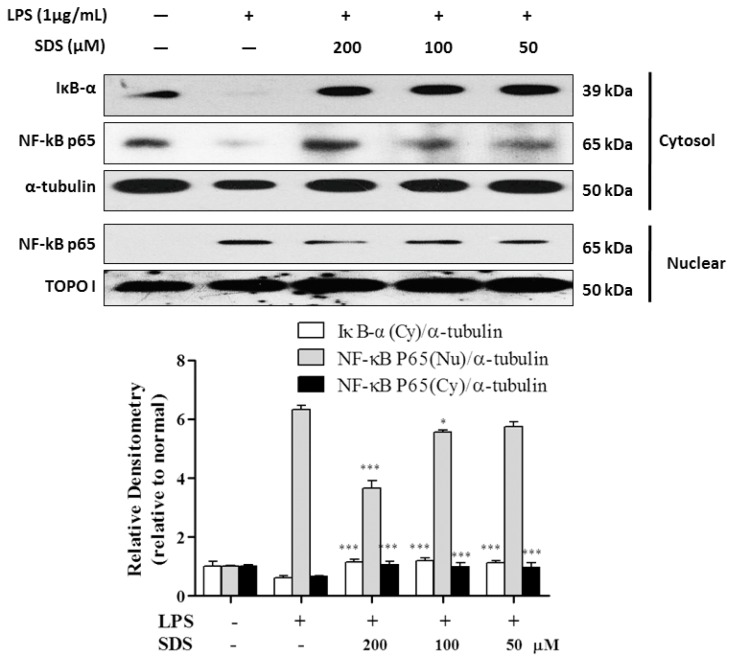
Effect of SDS on LPS-induced IκB-α degradation and NF-κB activation in RAW 264.7 cells. RAW 264.7 cells were pretreated with various concentrations of SDS for 1 h, followed by stimulated with LPS (1 μg/mL) for 30 min. The cells were harvested, and then protein was prepared for the detection of NF-κB p65 from in cytosol (Cy) or nuclear (Nu). α-tubulin (Cy) and Topo I (Nu) are loading controls of corresponding cellular fractions. Data are expressed as mean ± SD of three independent experiments. * *p* < 0.05, *** *p* < 0.001, versus LPS alone.

NF-κB is a key upstream regulator in the production of proinflammatory mediators in LPS-stimulated RAW 264.7 cells, such as iNOS and COX-2 [22]. Therefore, we next investigated whether SDS has an inhibitory ability on the NF-κB signaling pathway. As shown in Figure 4, LPS stimulation triggered the cytosolic IκB-α degradation and nuclear translocation of NF-κB p65 subunit. However, SDS pretreatment significantly attenuated the cytosolic level of IκB-α and the nuclear level of NF-κB p65 subunit, compared with LPS-primed RAW 264.7 cells. SDS exerted similarly anti-inflammatory effects when determining IL-1β and TNF-α mRNA being challenged with LPS.

**Figure 5:**
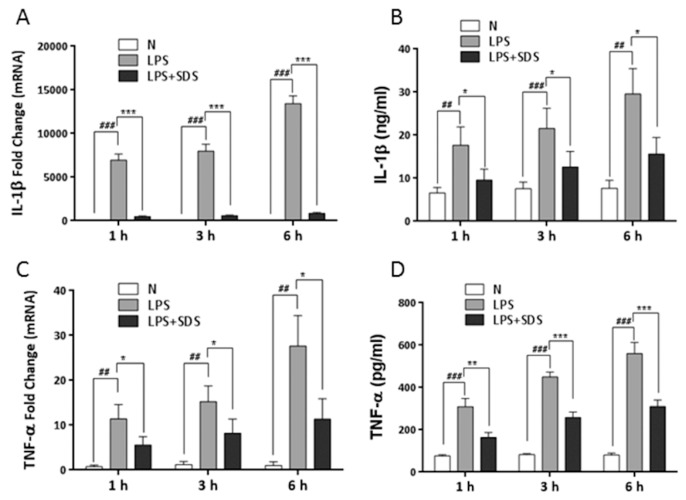
Effect of SDS on the synthesis and release of proinflammatory cytokines. RAW 264.7 cells were pretreated with SDS (100 μM) for 1 h, followed by stimulated with LPS (1 μg/mL) for 1, 3 or 6 h. mRNA expression of IL-1β (**A**) and TNF-α (**C**) was analyzed by real time PCR. Release of IL-1β (**B**) and TNF-α (**D**) in culture media was measured using ELISA. Data are expressed as means ± SD of three independent experiments. ^##^ *p* < 0.01, ^###^ *p* < 0.001, versus normal cells. * *p* < 0.05, ** *p* < 0.01, *** *p* < 0.001, versus LPS alone. N represents normal untreated cells.

Specifically, incubation of cells with LPS resulted in a significant time-dependent induction of IL-1β and TNF-α mRNA and reach peak at 6 h (Figure 5A,C). IL-1β and TNF-α in the cultured medium were detected within 6 h after stimulation with LPS by ELISA (Figure 5B,D). IL-1β and TNF-α levels time-dependently increased and also reach peak at 6 h. Application of SDS effectively suppressed LPS-induced mRNA expression and protein secretion of IL-1β and TNF-α in RAW 264.7 cells. It indicated that SDS might transiently inhibit the synthesis and secretion of proinflammatory cytokines induced by LPS.

### 2.6. Effect of SDS on Binge Drinking Induced Liver Injury

**Figure 6:**
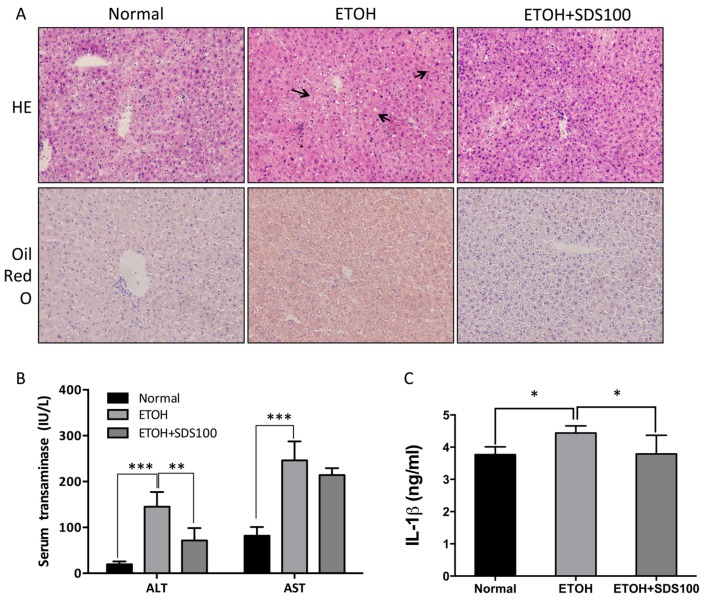
Effect of SDS on binge drinking induced liver injury. C57BL/6 mice were orally administrated SDS (100 mg/kg) for 5 days, followed by three gavages of dextrin maltose or ethanol every 30 min. (**A**) Representative H&E staining and Oil Red O staining of liver tissues; (**B**) serum ALT and AST; (**C**) serum IL-1β protein. Black arrows represent damaged hepatocytes. Values represent means ± SD. * *p* < 0.05, ** *p* < 0.01, *** *p* < 0.001, versus ethanol-fed group.

Binge drinking can create an inflammatory response in the liver, so we investigated the contribution of SDS to alcohol-induced liver injury in vivo in a model of acute alcohol intoxication caused by binge drinking. Alcoholic hepatitis is characterized by hepatocyte injury and inflammation. Mice receiving ethanol showed marked ballooning of the hepatocytes in the centrolobular area. The increased histologic findings of hepatocyte injury in alcohol-fed animals were associated with enhanced blood levels of AST and ALT (Figure 6A,B). The elevation in serum transaminase levels suggested the early stage of liver damage. SDS successively suppressed AST and ALT levels, as well as hepatocyte damage. As a result alcohol caused a marked increase in Oil Red O staining of lipid droplets. Pretreatment with SDS prior to alcohol ingestion significantly blunted alcohol-induced liver steatosis and enhanced transaminase levels.

IL-1β is a potent proinflammatory cytokine that is elevated in alcoholic liver injury [23]. IL-1β plays a pivotal role on the expression of proinflammatory genes by activation of intracellular signaling pathways, such as NF-κβ [24]. IL-1β was induced in mice fed ethanol, but it was greatly reduced in SDS pretreated mice, indicating the decrease of IL-1β may also impact hepatocyte survival.

## 3. Discussion

Inflammation is a host response to various stimuli that leads to the release of a large amount of inflammatory mediators [25]. Although SDS is known as regulating inflammatory responses via NF-κB activation in LPS-stimulated macrophages and LPS-treated mice, the detailed mechanism involving TLR4/TAK1 pathway remains unclear. To exploit the concept that SDS exhibited anti-inflammatory through blocking the endotoxin-induced expression of the TAK1 and thereby blocking the NF-κB signaling cascade in macrophages, firstly we determined the expression of TLR4, CD14, IL-1β, and COX-2 as well as iNOS protein levels and the release of NO in LPS-primed RAW 264.7 macrophages.

iNOS is induced by various inflammatory stimuli, including bacterial endotoxic LPS and inflammatory cytokines in macrophages. COX-2 is an inflammation-induced enzyme. At the sites of inflammation, it is highly expressed [26]. This suggested that iNOS and COX-2 plays a key role in inflammation. Our data showed that pretreatment with SDS caused a dose-dependent inhibition of COX-2 and iNOS at protein expression, which was consistent with previously reported literatures [20], confirming the anti-inflammatory effects of SDS.

In LPS signaling, LPS binds its major receptor CD14 and then initiate the intracellular signaling cascades. The interaction of LPS with LPS-binding protein (LBP) and its transfer to cellular CD14 activates macrophages [27]. Binding of LPS to Myeloid Differentiation 2 (MD-2) triggers the recruitment of the adaptors myeloid differentiation factor 88 (MyD88) and Toll-interleukin-1R domain-containing adapter inducing interferon-β (Trif) [28]. CD14 catalyzes TLR4-MD-2-LPS complex to initiate LPS-TLR4 signaling. Because TLR4 initiates the immune response to LPS, so we are intrigued whether SDS could block TLR4 signaling. As shown in Figure 2A, our data showed that protein expression of TLR4 and CD14 increased by LPS stimulation, while SDS successfully blocked LPS-TLR4 signaling pathway. These results suggest that inhibition of inflammatory mediators by SDS might be related with suppression of LPS-TLR4 signaling pathway. After TLR4 activation, TLR4-MD2 dimerization stimulates the intracellular pathway that eventually results in activation of NF-kB and the production of pro-inflammatory cytokines [29]. Considering SDS regulates activation of NF-kB in macrophages, we wondered how upstream of NF-kB affect the anti-inflammatory effects of SDS.

Upon LPS recognition, MyD88 in turn recruits IRAK4 and IRAK1, and then IRAK4 induces IRAK1 phosphorylation. Then phosphorylated IRAK1 leads to the phosphorylation of TAK1 [30]. TAK1 is a mitogen-activated protein kinase kinase kinase (MAP3K) and acts as upstream of NF-κB-inducing kinase (NIK) [31]. And NF-κB also regulates COX-2 and iNOS expression. Our result of increasing iNOS and COX-2 by LPS was accompanied with NF-κB P65 nuclear translocation. The activation of NF-κB requires phosphorylation of upstream IκB kinase (IKK) via upstream of TAK1. Our results showed that SDS pretreatment inhibited the increase in TAK1 phosphorylation firstly (Figure 3), and then eventually prevented nuclear translocation of NF-κB (Figure 4). And blockade of NF-κB activity with SDS leads to the inhibition of NF-κB target genes production, including proinflammatory cytokine, IL-1β and TNF-α (Figure 5). As mentioned in Figure 1B,C, SDS exhibited a slight but non-statistical reduction of RAW 264.7 cells viability at a high concentration in presence or absence of LPS within 24 h, especially at 200 μM, but there is no statistical difference compared to those of normal cells. So we selected a concentration with much lower potent cytotoxicity and much greater potent of inhibition ability against inflammation response.

MAPKs are responsible for the regulation of many genes involved in inflammatory mediators production. Upon LPS stimulation, JNK, ERK and p38 MAPK are activated via phosphorylation of both tyrosine and threonine residues and play different roles in different signaling capacities [32]. Our data revealed that SDS effectively blocked the signal transduction by MAPK molecules in LPS-stimulated macrophages (Figure 6), which was in line with previous report on THP-1 macrophages [33].

Acute alcohol consumption enhanced circulating levels of LPS in alcoholics with alcoholic liver diseases [34]. Therefore, during development of alcoholic liver disease (ALD), hepatic response to gut derived endotoxin (LPS) is a critical step [35]. Pre-exposure of alcohol can prime Kupffer cells to LPS stimulation, resulting in the increased release of proinflmmatory cytokines [36]. In regard to this, we applied the mice model of binge drinking-induced liver injury to mimic liver inflammation with hepatocyte injury to further investigate anti-inflammatory mechanism of SDS. Similar to the results obtained in vitro, SDS administration effectively alleviated alcohol-induced hepatocyte damage and proinflammatory cytokine secretion.

**Figure 7:**
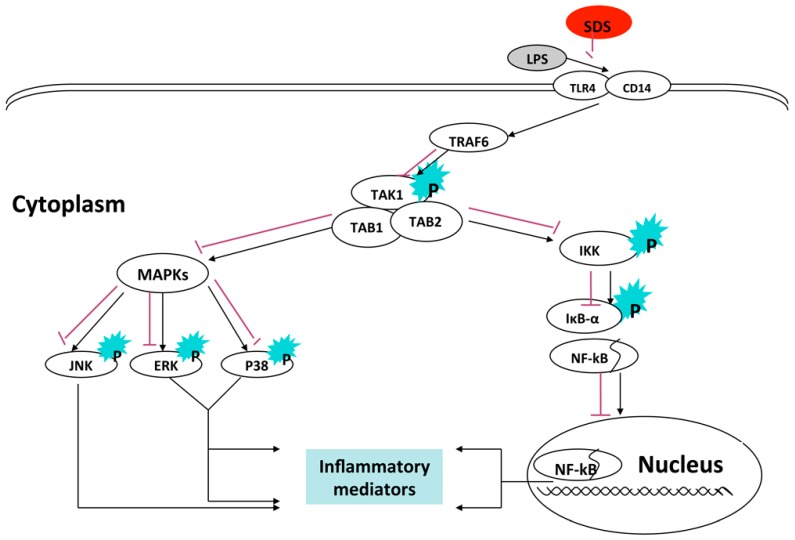
A proposed mechanism of anti-inflammatory effects of SDS.

In summary, this study demonstrated that SDS inhibited LPS-induced inflammatory mediators by attenuating the LPS-dependent induction of TLR4-CD14 signaling and inhibiting the subsequent phosphorylation of TAK1, and lead to inhibit NF-κB and MAPKs signaling pathway, eventually suppressing its proinflammatory target gene (Figure 7). Future studies will need to identify underlying mechanism responsible for beneficial effects of SDS in alcohol binge drinking induced liver inflammation, our results suggest that SDS may be therapeutically useful for the treatment of hepatic inflammation associated with an activation of TAK1.

## 4. Materials and Methods

### 4.1. Materials

SDS (99%) was obtained from National Institute for the Control of Pharmaceutical and Biological Products (Beijing, China). All cell culture reagents were from Gibco (Stockholm, Sweden). Lipopolysaccharide (LPS) and dimethyl sulfoxide (DMSO) were purchased from Sigma Chemicals Co. (St. Louis, MO, USA). All reagents were purchased from Beyotime Institute of Biotechnology (Nantong, China). Anti-iNOS, anti-p-TAK1, anti-P-JNK, anti-P-ERK and anti-P-p38 antibodies were purchased from Cell Signaling Technology (Danvers, MA, USA). Anti COX-2, anti-CD14, anti-TLR4, anti-NF-κB P65, anti-IκBα and anti-topoisomerase I were purchased from Santa Cruz Biotechnology (Santa Cruz, CA, USA) and anti-α-tubulin was purchased from Sigma.

### 4.2. Cell Culture

RAW 264.7 cells, a mouse macrophage cell line, were cultured in Dulbecco’s Modified Eagle Medium (DMEM) medium containing 10% fetal bovine serum, 100 U/mL penicillin, 100 μg/mL streptomycin and incubated at 37 °C in 5% CO_2_.

### 4.3. Measurement of Cell Viability

After pretreated with different concentrations of SDS (0–200 μM) for 1 h, RAW 264.7 cells were followed by stimulations absence or presence of LPS (1 μg/mL) for 24 h. Subsequently cells were incubated with MTT solution (5 mg/mL) for another 3 h. Then the content of formazan was measured in DMSO at 570 nm using a microplate reader.

### 4.4. Quantification of NO Production

The nitrite accumulated in medium supernatant was measured as NO production based on the Griess reaction as described previously [27].

### 4.5. Binge Drinking Protocol

Twenty week-old male C57BL/6 mice were obtained from Animal Division of Jilin University (Changchun, China). Procedures were carried out according to the criteria of the “Guide for the Care and Use of Laboratory Animals” published by the USA National Institutes of Health and approved by Animal Research Committee of the University. Animals were maintained at ambient temperature and humidity under an artificial 12 h light/12 h dark cycle.

Mice were randomly divided into three groups: normal group, ethanol group and ethanol plus SDS group. Mice were fasted for 4 h and then received three gavages of equivalent calories of dextrin maltose or ethanol (3.5 g/kg) every 30 min, and then mice were sacrificed 6 h later [37]. Before gavaging ethanol, the mice in ethanol plus SDS group were orally administrated SDS (100 mg/mL) for five continuous days. The doses of SDS were determined according to our previous experiments [17,38]. Livers were harvested, snap frozen in liquid nitrogen, and stored at −80 °C until they were used. 

### 4.6. Blood Biochemistry and ELISA Assay

Serum levels of alanine aminotransferase (ALT) and aspartate aminotransferase (AST) were detected using an Autodry Chemistry Analyzer (SPOTCHEM^TM^ SP4410, Arkray, Kyoto, Japan). Serum IL-1β levels were determined by Murine IL-1β Standard ABTS ELISA Development Kit (PeproTech Inc., Rocky Hill, NJ, USA) according to the manufacturer’s protocol. Release of IL-1β and TNF-α in culture media of RAW 264.7 cells was measured using ELISA Development Kit (PeproTech).

### 4.7. Real-Time RT-PCR

RNA was isolated from cells using by using SV Total RNA Isolation System (Promega, Madison, WI, USA) according to the manufacturer's instructions. RNA (500 ng) was reverse transcribed using Oligo(dT)15 Primer, RNasin Ribonuclease Inhibitors (Promega) and AMV reverse transcriptase (Promega) according to the manufacturer's instructions. Relative gene expression was determined by real-time PCR with FastStart SYBR Green Master (Roche) on an Agilent Mx3000P QPCR System using the comparative ΔΔCt method and GAPDH as the housekeeping gene. The following primers were used for analyses of gene expression in murine RAW 264.7 cells: mIL-1β-forward, 5′-TCTTTGAAGAAGAGCCCATCC-3′; mIL-1β-reverse, 5′-CTAATGGGAACGTCACACAC-3′; mTNF-α-forward, 5′-ATCAGTTCTATGGCCCAGAC-3′; mTNF-α-reverse, 5′-TCCACTTGGTGGTTTGCT AC-3′; mGAPDH-forward, 5′-CTTGTGCAGTGCCAGCC-3′; mGAPDH-reverse, 5′-GCCCAATACG GCCAAATCC-3′.

### 4.8. Histochemistry

Livers removed from mice were rapidly frozen in optimal cutting temperature (OCT) medium and were stored at −80 °C until use. Serial sections of 5 μm were cut and were fixed in an iced mixture of acetone–methanol (*v*:*v*, 1:1). The hematoxylin and eosin-stained sections were evaluated by light microscopy. Lipid accumulation was detected using Oil Red O staining as described by Kanuri et al. [39].

### 4.9. Western Blotting Analysis

After pretreated with various concentrations of SDS for 1 h, RAW 264.7 cells were stimulated with 1 μg/mL LPS for 24 hours for iNOS, COX-2, CD14 and TLR4 detection. For the detection of NF-κB p65, IκB-α, p-TAK1, p-ERK p-JNK and p-p38, the cells were preteated with various concentrations of SDS and then subsequently stimulated with LPS for another 30 min. Whole cell lysates were prepared using ice-cold cell lysis buffer (Beyotime, Nantong, China). Nuclear and cytosol fractions were prepared from cells using the reagent-based method (Beyotime). Protein concentration was determined using BCA Protein Assay Kit (Beyotime).

Cell lysates were separated by 10% SDS-polyacrylamide gel electrophoresis and electrotransferred to polyvinylidene difluoride (PVDF) membranes. The membrane was incubated in blocking solution (5% nonfat milk in PBS with 0.1% Tween 20) at room temperature for 1 h, and then incubated overnight at 4 °C with specific primary antibodies, followed by incubation with horseradish peroxidase (HRP)-conjugated secondary antibodies (Santa Cruz Biotechnology) for 1 h at room temperature. Immunoreactive protein was visualized by the BeyoECL plus kit (Beyotime). To correct for potential unequal loading, blots were stripped and reproved with antibodies to topoisomerase I (Topo I) for nuclear fractions and α-tubulin for cytoplasmic fractions. The relative intensity of each band was quantified by densitometric analysis using Quantity One software (Bio-Rad, Hercules, CA, USA).

### 4.10. Statistical Analysis

All data were expressed as mean ± SD. Data analysis was performed with one-way ANOVA and Tukey’s multiple comparison tests by using the GraphPad Prism program (GraphPad Software, San Diego, CA, USA). Statistical significance was defined as *p* values less than 0.05.

## 5. Conclusions

The suppression of LPS-induced inflammatory mediators by SDS is mediated by inhibition of TAKI phosphorylation, leading to inhibition of NF-κB and MAPK phosphorylation, eventually suppressing its proinflammatory target gene.

## Abbreviations

The following abbreviations are used in this manuscript:

ALT	alanine aminotransferase
AST	aspartate aminotransferase
ERK	extracellular signal regulated kinase
iNOS	inducible nitric oxide synthase
COX-2	cyclooxygenase-2
IκB-α	inhibitory κB-α
IKK	IκB kinase
IRAK	interleukin-1 receptor-associated kinase
JNK	c-Jun terminal kinase
LPS	lipopolysaccharide
LBP	lipopolysaccharide-binding protein
MAPK	mitogen-activated protein kinases
MD-2	myeloid differentiation-2
MyD88	myeloid differentiation primary response gene 88
NF-κB	nuclear factor-κB
TAK1	TGF-β activated kinase 1
TLR4	toll-like receptor 4
TRAF6	TNF receptor associated factor 6
TRIF	toll/IL-1 receptor domain containing adaptor inducing interferon-β
